# The experience of pre-hospital emergency personnel in breaking death news: a phenomenological study

**DOI:** 10.1186/s12912-022-00899-x

**Published:** 2022-05-25

**Authors:** Reza Safari, Mohammad Mehdi Khashmin, Alireza Abdi

**Affiliations:** 1 Student Research Committee, Kermanshah University Of Medical Sciences, Kermanshah, Iran; 2 Lecturer of Nursing, Emergency and Critical Care Nursing Department, Nursing and Midwifery School, Kermanshah University of Medical Sciences, Kermanshah, Iran; 3 Associate Professor of Nursing, Emergency and Critical Care Nursing Department, Nursing and Midwifery School, Kermanshah University of Medical Sciences, Kermanshah, Iran

**Keywords:** Emergency, Pre-hospital, Death, Qualitative study

## Abstract

**Background:**

Today, breaking the death of patients to their families has become one of the challenges for medical staff. Considering the lack of study in the pre-hospital emergency, the present study aimed to explore the experience of pre-hospital emergency personnel regarding the breaking death news to families.

**Method:**

In this qualitative study with a descriptive phenomenological method, data were collected by purposeful sampling method through in-depth interviews with thirteen pre-hospital emergency personnel in Kermanshah and Kurdistan provinces. After recording and writing the interviews, the data were managed by MAQUDA-10 software and analyzed using the Collaizi approach.

**Results:**

Of 13 participants, five from Kermanshah, eight from Kurdistan, and 12 (92%) were married. The mean age and work experience were 34.38 and 10.38 years, respectively. Five main extracted themes were 1) perceived stress, 2) challenge factors of breaking death news, 3) unnecessary actions, 4) death breaking precautions, and 5) BDN requirements. They were covered fifteen sub-themes.

**Conclusion:**

In this study, emergency medical employees were always faced with stress and challenges to announce the patient's death to families, including the stress of violence against employees. Hereof, personnel had to take unnecessary care actions such as slow resuscitation to transfer the patient to the hospital.

**Supplementary Information:**

The online version contains supplementary material available at 10.1186/s12912-022-00899-x.

## Background

Breaking bad news to families has subtle psychological details and is always a challenging process for healthcare workers [1]. Many of the person's emotional reactions are closely related to the environment, culture, and religious beliefs [2], likewise, in some medical centers, corpses are placed in a specific position for moral considerations [3]. Presenting bad news as a job difficulty has been accompanying the medical profession from the beginning [4], and has not received much attention until the last half-century [5]. Even its accepted definition is returned to the 1980s. According to this definition, "bad news is news that causes a strong emotional reaction, unpleasant behavior and feelings in the listener and a bad way of looking at the future" [6]. From a philosophical view, the nature of bad news is not good or bad, it is the feeling that is created in the listener of the news that makes it bad [7]. Therefore, the speaker can realize the bad news only after giving the news and observing the listener's reactions [8].

Breaking bad news (BBN) is a worrying activity for physicians and medical staff. As a result, the resulting stress has created challenges in the process of therapeutic care, which causes psychological stress among healthcare workers [9]. This situation pulls out the staff from clinical duty to humanity and is considered an ethical challenge for them. EMS personnel, because of time restrictions and lack of preparation, could not use the defined frameworks for BBN such as SPIKE and ABCDE models, therefore, they are in danger of failure and additional stress [10]. This situation is also difficult and anxious for patients and their families [10]. likewise, a precise, step-by-step, and modified action plan is needed to break the bad news, reduce grief based on moral principles, and consider the families' reactions to announcing the death of their patient [11]. In general, having a strategy to announce a death or bad news to families is better than stopping it or telling it in a reckless manner [12].

Pre-hospital emergency personnel usually have to report bad news in a situation where they have the least possible time to be familiar with the recipient and are often unaware of the root cause of the bad news they have to report [13]. Also, despite the frequent need to announce the bad news and the death of the patient to the companions in the emergency department, not many studies have been done in this field, furthermore, generalization of the results of studies conducted in non-emergency departments to the emergency wards is impossible [3]. While non-emergency departments usually have ample opportunity to prepare the condition of the attendants before BBN. However, there is not enough time and a suitable physical environment to provide services in the EMS [14].

Considering the lack of defined and legal guidelines for BBN in Iran, and the lack of information about EMS, the present study was conducted for exploring the experience of pre-hospital emergency personnel in breaking death news (BDN) in a qualitative study.

## Methods

This qualitative research was conducted in descriptive phenomenology [15] from September 2020 to June 2021. We applied the COREQ checklist as the guide for the project [16].

### Participants

the participants were the staff of emergency medical service (EMS) of Kermanshah and Kurdistan provinces of Iran, who were recruited as the purposeful sampling. Inclusion criteria consisted of having consent to expressing their experiences about BDN, having at least two years of work experience in EMS, and having at least one time involved in BDN with families. Written informed consent was obtained from the participants for recording their voices and participation in the research.

### Data collection

We utilized in-depth face-to-face interviews to collect data. To accomplish this process, at first demographic information such as type of employment, work experience, place of work, and education degree were taken. Some open-ended questions had been asked and followed by probing questions. For example, questions such as " How are your experiences with breaking death news to families?" and elaborated with probing questions such as, “why, how and give me an example". We wrote the important notes for conducting the interviews. The criterion for finishing the sampling was based on data saturation and the absence of new data and themes, which was met in interview twelve.

Data collection was started with the EMS personnel of Kurdistan by the first researcher (Reza Safari, MSc student). Each interview was recorded by cell phone of Samsung Galaxy A8, and immediately typed verbatim on the same day, and the analysis was commenced by Reza Safari and Alireza Abdi (associate professor of nursing and expert in qualitative research). Before interviewing the participants, written informed consent was taken from them to participate in the study and record their voices, next, they were asked to conduct interviews in a place where it was easier for them to prevent disruption. So that we accomplished the interviews at the participants' workplace on the previous agreement. Moreover, before each interview, we explained the topic and aims of the research. Interviews lasted about 40–60 min (on average 45 min).

### Data analysis

Data analysis was fulfilled based on the seven-step approach of Collizzi which has been enlightened by Morrow et al. and Shosha [17, 18], which is managed in MAXQUDA-10® by VERBI Software GmbH (Berlin, Germany) software. In the first stage, we listened to the recorded interviews repeatedly several times, then wrote them verbatim, and the written interview was read several times. Consequently, the meaningful statements related to the phenomenon (BDN) were identified. In the second stage, after reading all the participants' descriptions, the meaningful information was underlined. The third step was continued by extracting the important phrases (Formulated meanings) and an attempt was made to extract a representative concept (code) from each phrase. After obtaining these compiled concepts, an attempt was made to examine their relevance to the initial sentences and to ensure their correctness. After extracting the codes, according to the fourth step of Collaizi, the researcher carefully studied the assembled concepts and categorized them into sub-themes based on their similarity. In the fifth step, the results were interconnected for a comprehensive description of the phenomenon and we created more general themes. In the sixth stage, a comprehensive description of the BDN was scrutinized as clearly and unambiguously as possible. The final step was to check the trustworthiness of the data.

### Trustworthiness

Rigor was held on Lincoln and Guba's approach [19], for credibility, we were involved in the data collection for six months, and variation in participants' characteristics in terms of age and work experience was maintained. Codes and themes were also provided to the participants (member check) and co-workers (peer check) and after addressing their comments, approved by them. For dependability, we elaborated the collecting process of data, analysis, and extracting themes, so they can be properly audited by external observers. In line with confirmability, the researcher tried to extract the codes and themes from the participants' descriptions and suspend his previous knowledge. For transferability, the phenomenon of BDN was summarized and the results of the study were provided to three EMS personnel who did not participate in the study and their experiences were commensurate with our results.

### Findings

In this study, thirteen EMS personnel from Kermanshah and Kurdistan provinces participated, 12 (92%) were married and 8 (61.5%) had a bachelor's degree in emergency medicine. The mean (SD) of age was 34.38 (2.53) years with a domain of 30–39 years, and work experience was 10.38 (2.56) years with a domain of 6–15 years (Table 1).

**Table 1:** Demographic characteristics of the participants

Medical urgency staff	Marital status	Age	Education qualification	work experience in EMS (years)
P1	Married	34	Bachelor of Medical Emergency	10
P2	Married	33	Bachelor of Medical Emergency	10
P3	Married	36	Bachelor of Medical Emergency	10
P4	Married	37	Associate’s Degree of Medical Emergency	11
P5	Single	30	Bachelor of Medical Emergency	6
P6	Married	38	Bachelor of Anesthesia	15
P7	Married	33	Bachelor of Medical Emergency	8
P8	Married	39	Associate’s Degree of Medical Emergency	15
P9	Married	33	Bachelor of Medical Emergency	8
P10	Married	34	Master of Emergency Nursing	9
P11	Married	35	Master of Medical Education Technology (with Bachelor of nursing)	12
P12	Married	33	Bachelor of Medical Emergency	10
P13	Married	32	Bachelor of Medical Emergency	11

From the qualitative content analysis, 680 codes, five main themes, and fifteen sub-themes were extracted which are presented in Table 2.

**Table 2:** Themes and sub-themes extracted from the study

Main themes	sub-themes
1.Perceived stress	1) Violent stress
	2) Family health stress
	3) non-acceptance stress
2.Challenging factors of breaking death news	1) Individual characteristics
	2) accident site
	3) Type of death
	4) Postpone BDN to hospital
3.Unnecessary actions	1) futile works
	2) slow code
4.Death breaking precautions	1) Ensure scene safety
	2) Assessing the conditions
	3) Scene-management problems
4.BDN requirements	1) public education
	2) Staff training
	3) Death breaking experience

#### Perceived stress

The concept of perceived stress was one of the main themes in this study. Most participants were exposed to the stress of entourage violence, others had the stress of the patient's family health, and some employees also talked about not being accepted by their companions. In this regard, three sub-concepts 1) violence stress, 2) family health stress, and 3) denial stress, were obtained.

#### Violence stress

Violent stress was extracted based on the views of all the participants. Fear and stress of violence from the deceased's companion and family made pre-hospital emergency staff always at risk of being beaten. Violence perpetrated by companions sometimes disrupted services for patients and left them unable to focus on treatment, and the staff was forced to delay treatment until police arrived.

participant 1, about confronting a stressful situation, stated:

“The only way I could think of it (breaking death news) was to postpone the death notice when we arrived at the hospital to announce the doctor and inevitably started CPR,… because I knew that in the hospital environment, despite the security forces and other medical staff, there was no danger of violence to me”.

This violence sometimes caused damage to ambulances and other equipment, and the stress of further violence caused the death declaration of the deceased to be postponed until the police arrived in the area. Some participants complained that police and other staff who should be immediately present at the scene did not have the necessary cooperation with them and would come too late.

"In missions where there is a suspicious death, such as suicide or altercation, before doing anything, the police must be present to make the scene safe for us in every way, and we must not be afraid of harming ourselves or our ambulance. “. (Participant No. 7).

#### Family health stress

Some of the participants had indicated increasing in anxiety and stress of family members after BND. EMS personnel would be unable to make a good death notice if they were likely at risk to the family's health after hearing the death news at the scene, and some of them could not prevent the family's health from being compromised. So, the BDN was postponed to another location.

Participant 1 stated:

"In one case…, we were transferring a middle-aged man who was hit in the head and suffered a cardiac arrest on the road. After a few minutes of CPR, I told his two sons that the patient did not have a pulse, he died. But the sudden reflection of the news was that one of his sons hit his head in the ambulance cabin."

In cases where there were one or two family members, and it needed a long time for other family members to arrive, the participants preferred to transport the deceased to a hospital or morgue so that those individuals would not suffer more from this psychological damage.

"I did not want that family to cry and mourn when they saw their father's body until the morning, that is, I was personally concerned about the health of the rest of the family."(Participant No. 9).

#### Denial stress

Participants believed that factors such as "loss of the mainstay” and lack of families' confidence in the expertise of pre-hospital staff, had worsened the acceptance of death.

participant 2 stated:

"The acceptance of death by the family goes back to their understanding of death, which means how much they accepted it because some of the family refuses to accept that they lost their loved one…At that moment, the behavior of families is not normal at all, especially those who have lost the main vertebrae (means parents) of the family."

Some families expect to hear good news or even miracles, which caused technicians to fail to break death.

Participant 8 stated:

"Normally, we should measure the acceptance of the family to hear the news of death because some of them expect you to resurrect their loved one and this is why they do not accept death or deny it."

Some participants believed that inappropriate family behavior following the BDN of sudden death was a natural process and the family should be given the right to do it.

### Challenge factors of breaking death news

This concept was another major theme in this study. According to the interviewees, the staff responsible for announcing the death had experienced many behavioral and stressful challenges. In this regard, four sub-theme were obtained: 1) individual characteristics, 2) accident site, 3) type of death, and 4) Postpone BDN to hospital.

#### Individual characteristics

Most participants believed that the BDN was different in pre-hospital conditions and that was not difficult to declare it in people with characteristics such as a history of chronic disease, addiction, and old age, but it was much harder against people who were in good health or a person whose family did not expect to die.

Participant 1 announced:

"In children, young people, and mothers with young children, we have a lot of stress and anxiety at the time of their death announcement, and we often fail".

"We had a mission to be on the side of an elderly person who had been at bed for several years and was a slag for himself and his family, I had no concerns about announcing the death" (Participant No. 13).

Some personnel commented that because of differences in the behavior of the families, they see different reactions from them, and it is not possible to say at the point which reaction is specific to which type of death.

Participant 13 stated:

"One mission that was very surprising to me was that a newborn had died and we would have presented at the scene. We arrived, her mother was present above her head, but she wasn't too upset and said I breastfed her, which jumped into her throat and choked... Later, we heard from the police we were in contact with, that the mother has strangled (the neonate) herself because of postpartum depression and caused her death."

#### Accident site

Participants stated that the place where the person died is another important challenge in announcing the death of the companions. They believed that informing their companions of the death at road sites was more difficult for them than urban operations due to a large number of companions present at the scene. Some of them also stated that the presence of non-specialist forces in the dispatch unit, who may have made incorrect decisions about how to deliver the operational unit to the desired location, caused delays in reaching the location and increased the workload of pre-hospital emergency personnel.

Participant 6 articulated:

"Sometimes in the car accident missions we go to, we see that the family of the deceased is present at the scene, and one of the most challenging types of missions in this condition is that you want to announce the death of a loved one, and when you see that the companions are very restless, it is very difficult to announce their death. Because most of the time there are other injured people on the scene and there is a lot of pressure on the staff to announce the death of someone."

#### Type of death

Participants declared that how the person died was an important challenge in displaying death to the companions. In other words, the deaths of the participants were divided into two categories of sudden deaths and predictable deaths, each of which had its different challenge for the personnel and stated that those who had sudden death were usually people who had sudden death due to accidents, trauma or unexpected events, and their families were not expecting their deaths, whose death announce led to problems such as shock and denial. However, the deaths of people who were not in good health or were older were not accompanied by many challenges and the ‌‌‌BDN was easier.

Participant 13 said:

"We had another mission recently. We went and there was an old man who had died of old age. When we examined him, it was clear that (it passed) a few hours after his death and even irreversible, and there was no or much less stress and anxiety about announcing death”.

Participant 3 displayed a difficult case for breaking death news:

“On a mission near our base, a child with a construction hoist fell on his body and we immediately transported him to the ambulance, but when I checked his vital signs, he had nothing and died.”

#### Postpone BDN to hospital

Some participants believed that in missions where the scene of the accident was turbulent and unstable, breaking news in another quieter place was more acceptable for families. Some participants also believed that BDN to family members in an environment other than the scene of the accident (such as the hospital) could be tolerable because of the satisfaction of all the actions taken. They declared that because they consider the hospital to be the end of the line, or that in addition to the presence of law enforcement forces to ensure the safety of personnel, it can also hand over responsibility for transferring this news to the medical or nursing staff.

Participant 3 stated:

"My suggestion is that in some cases where there is a fear of injury or beating, we can transfer the deceased to the hospital because of the protection force and the fear of self-harm and ambulance is much less, secondly because there are other doctors and nurses there, it is easier to announce death."

"If the environment is violent and I and my equipment are (likely) damaged, rest assured, I will never announce the death and, based on my own experience, I will transfer the dead (patient) to the hospital to protect myself and my colleague and delegate the dying declaration to the hospital staff." (Participant No.4).

### Unnecessary actions

Unnecessary actions were another major theme in this study. The findings confirmed that the participants did not consider unnecessary actions as part of their responsibilities and duties. But companions often force them to take unnecessary actions because at that moment they are the only rescuer available to the family. In this regard, two sub-themes (1) futile works and 2) slow code, were obtained.

#### Futile works

Participants found it is ineffective to treat a person long after death. In some cases, participants complained of inappropriate interference, arguing that forcing staff to do some actions that waste the staff's time, and another client might need a pre-hospital emergency team at the same time.

"In the scene, some families were very aggressive and restless, and we had to (unnecessarily) take that dead person to the hospital,” (participant No. 6).

Participant 12 also noted over a mission with a dead person:

"We pretended to examine the heart-lung audition, and while we knew it was of no use."

"Sometimes we see families forcing us to do things that are not our job at all. Unreasonable interventions or unrealistic expectations increase the burnout of us and other co-workers."(participant No.9).

Due to the deceased condition, sometimes it was necessary to tell expedient lies, as a futile action, to the patient's companions to protect against family tension.

“for fear of being beaten, I had to tell them that your father was not dead and still had a pulse." (participant No. 1).

However, some participants did not agree with telling an expedient lie about the deceased's condition, believing that lying to the family about the client's condition would have worse consequences.

#### Slow code

According to the participants, the slow code performed by the personnel is another unnecessary measure in this regard.

Participant number 6 stated:

"At the insistence of the family, we did nearly half an hour of CPR while we knew that person had died and nothing could be done for him."

Some EMS personnel remarked that they were forced by companions to perform a show resuscitation to convince the family that they had done their best to revive the dead person.

participant 3 stated:

"Trying to communicate well with that person (the companion), like, let’s help (me to do some works), he understands that you're doing everything you can to bring that loved one to life, and then if you find out he's not coming back, do some kind of show resuscitation to get him to the hospital."

### Death breaking precautions

This concept was another main subject in this study. It conveys that special measures and precautions that vary in different circumstances, should be considered for BDN. Paying attention to these issues reduces the rate of violence against personnel and better acceptance of this by deceased companions. In this regard, 3 sub-themes 1) ensuring the safety of the scene, 2) assessing the conditions, and 3) Scene-management problems, were obtained.

#### Ensuring scene safety

Participants stated that ensuring the safety of the scene is the principle that should be taken into consideration before entering the missions and even during treatment. This led them to accomplish BDN with greater safety and caution. Some personnel need minimum stress to comply with safety principles when breaking death news.

participant 5 stated:

"The safety of the scene is very important for us because we need to assess what scenes the family is prepared to hear about the death and what scenes do not meet the conditions for accepting death."

Also, participant 13 noted:

"I will definitely consider the principles that examine the safety of the scene, which includes the safety of the personnel themselves, the safety of the scene, the overall investigation and the investigation of the conditions, then I decide to declare or not the death of the patient."

#### Assessing the conditions

Participants considered the family circumstances as a general perception of the reaction and behavior of family members before and after the BDN. Evaluation of deceased family conditions is a dynamic and continuous action and can be effective on death declaration. They believed that by speaking with those people and communicating properly, it is possible to understand their reaction after hearing the news of death. They also stated that in the missions that informed the death at the scene, expressing sentences such as,

"We will do everything we can, you pray for your loved one" (Participant No. 7), will help them in BDN to the deceased family.

Regarding the assessment of the family's conditions before the announcement of the death participant 1 stated:

"By assessing the circumstances of the family how they reacted and to what extent they coped with their loved one's death, whether they were a quiet and calm family or not, I would have decided to declare death..., and before saying it, we need to have a full view of the circumstances of that family to see how far they accept death and come to terms with it that if that's the case, we'll gently prepare their minds to hear the news of death."

#### Scene-management problems

Communication problems and barriers caused by the family and companions of the deceased person had caused the pre-hospital emergency workers to be unable to be successful in announcing the death and managing the scene. Some colleagues were unable to articulate properly with the deceased family due to lack of experience, which was later left in their minds as an unpleasant experience, and believed that scene management power can be achieved after experiencing several death notices. Participant 6 concerning a road accident mission involving two deaths and four injured people, after insisting that the deceased be transferred and failing to declare their deaths, stated:

"I couldn't manage the scene well and I didn't dare to announce the deaths of those two people to the entourage, and now, 12 years after that incident, I still have it in my mind and every time that scene happens to me, I feel a kind of fear that how I can manage the scene well."

In this regard, participant 5 stated,

"The early days (the initial time of employment) when I was going to manage the die patients, maybe because of the lack of experience, I had said sudden declaration that made the family restless and did unreasonable things."

### BDN requirements

The concept of BDN requirements was another main subject in this study. Participants believed that it is not easy for anyone to announce a death, especially when people in the community have not been taught any training about understanding death and controlling emotions and behavior after hearing it. In this regard, three sub-themes 1) public education, 2) staff training, and 3) BDN experience, were obtained.

#### Public education

Most EMS personnel stated that to better manage the BDN, education through schools and mass media should be planned, through which they should improve people's view of pre-hospital emergency tasks. Some believed that social culture in cooperation with the pre-hospital emergency unit was one of the requirements for managing BDN.

Participant 10 on the importance of education and the positive effects on the general public stated:

"To increase the knowledge and awareness of society, education speaks first because when people in a society are aware of the ability and function of emergency 115 (the phone of EMS in Iran), it is much better to announce death to them and they will accept it in a way because they are familiar with the process of death, but unfortunately this is not taught in our country at all. or there's very little training."

Regarding the challenges in the field of public education, participating 9 indicated:

"We have no training that increases the public's understanding of the pre-hospital emergency system, and this lack of awareness itself has a lot of consequences, which is the main burden of the 115 (EMS) personnel, so please, our authorities are expressing ways to increase the understanding of the community either through the media or through other training."

#### Staff training

Participants remarked that staff training should be regarded to manage death declarations. Some believed that death notification should be developed in academic courses by compiling the headlines of death announcements and continuous education into the curriculum of EMS for increasing their skills.

Participant 9 stated:

"There is no workshop for us at all to show us the way and how to talk to the deceased family and somehow complement the teachings of the student course."

Others also believed that creating retraining courses as well as holding workshops related to death notices could be very helpful. Some participants also believed that they had been traumatized after missions with several deaths, which suggested referring to a psychologist after completing their mission. In this regard, participant 2 stated:

"Unfortunately, the retraining in Iran is very simple, especially for pre-hospital emergency colleagues and due to the lack of psychology classes for personnel and adequate training, I think the personnel must refer to a psychologist to be mentally prepared to act successfully in the announcement of death."

#### Death breaking experience

Almost all EMS personnel believed in the role of clinical experience in the field of death declaration and stated that personnel who are experienced in this field can better declare death to families and this experience was achieved by engaging with many missions that lead to death notices. In this regard, participant 5 stated:

"The death notice requires someone who has the necessary work experience who can announce death to the family very well."

If EMS personnel were responsible for ‌BDN to the family, they used tricks such as introduction and morale to the family. One participant explained how to deliver the news of the death:

"My goal was to start telling them that their father (as the patient) had passed away and even explained to them that when we do CPR, it means that the person has no pulse and breathing, and we're trying to revive him" (participant No. 10).

## Discussion

### Perceived stress

This study was conducted to explore the experience of pre-hospital EMS personnel about the breaking of the patient's death to the families. The first theme extracted is "perceived stress", which emphasizes the possible harm caused by dealing with stressful environmental experiences that are inflicted on personnel. Studies show that most medical staff experience verbal and physical violence from patients' companions [20]. Also, verbal abuse and threat were the most common among nurses, and physical, sexual, and racial violence had the lowest statistics [21]. In 2018, Emam et al. stated that verbal violence was the most common type of violence against nurses and that patients' companions were the most common source of violence [22]. Breaking bad news is considered one of the main reasons for violence against health care workers, mostly against emergency departments personnel, with disastrous consequences for staff (frustration and burn-out), patients, and organizations [23–25].

Another issue that caused stress in the staff was the risk to the health of families after receiving the death news. In a study, patient death was the most concern and stress of the family [26]. Other studies also believed that some physicians, due to concern for family health, and potential medical issues, preferred that relatives not be present during CPR and introduced this action as a strategy to reduce family health stress [27–29]. However, this action may be effective in BBN for the family with dead children [30]. Regarding the possibility of non-acceptance stress and the possible reaction of the entourage, Taylor et al. 2007 believed that relatives' reactions to bad news could not be predicted. They can express reactions such as anger, tears, denial, verbal abuse, threatening behavior, and silence [31]. The Royal College of Pediatrics in London has also stated that personnel should be careful of a range of reactions after hearing distressing news from their families and that they may not accept the death of their loved ones [32].

### Challenge factors of breaking death news

The concept of challenge factors affecting the BDN was another major issue in this study. The individual characteristics of the deceased person were more challenging for employees to give BDN to their companions, including the death of a pregnant mother or a child than the deaths of other people. Dartey et al. 2019 also, in a qualitative study expressed acceptance of maternal death as a major challenge and problem [33]. Almeida et al. also revealed that the death of a newborn baby within hours or days after birth may seem catastrophic because during pregnancy, expectations, and dreams of having a son/daughter are imprinted in the minds of families [34]. Clark et al. noted that prolonged death allows news recipients to gradually realize that their loved one's death is happening as doctors and nurses prepare them through successive news stories [35]. In the viewpoint of Mainds et al., while in-hospital and out-hospital CPRs are clinically the same, both have different challenges. Out-hospital is very unpredictable with factors beyond the control of paramedic staff. The incident location, the number of people present at the scene, the age of the family, and the patient's condition [36] are some of these factors. The place of death is considered one of the most important aspects of end-of-life care. Ordinary people, health care policymakers, and providers place different importance on the place of death [37].

### Unnecessary actions

The third main theme in this study was unnecessary actions. Unnecessary measures are likely to increase personnel burnout and impose additional costs on the treatment system. In this regard, Hakkler et.al. stated that there is no need for futile CPR to be offered to patients or at the request of families because an action is useless and does not have any desirable physiological effect [38]. In addition to burnout, unnecessary measures can result in additional costs. In this regard, Sok et al. (2020) stated that continuing treatment of people whose treatment is useless may take the opportunity to use other patients for hospitalization, resulting in unnecessary waste of medical resources, inefficient distribution of resources, and imposing double costs on the treatment system [39]. However, family's choice of CPR should be respected "unless it's useless. And physicians should not provide services that are deemed unnecessary or useless [40]. The conditions of prehospital care are different, while unnecessary actions are an ethical challenge, identifying the futility of work is difficult in this area, however, these works signal to the families and companions that everything possible has been accomplished for the dead person, which facilitate the grief process [41].

### Death breaking precautions

The concept of death-breaking precautions was another main subject in this study. It represents special measures and precautions that vary in different circumstances. Paying attention to these issues reduces the rate of violence against personnel and better acceptance of this by the deceased's companions. In the 2020 heart protocol, ensuring scene safety is considered essential as the first step for CPR [42]. The resuscitation guidelines have always emphasized that staff safety and that resuscitation team members are the number one priority when trying to resuscitate [43]. The most important point is for nurses to ensure their safety and follow appropriate advice and guidance for personal protection equipment and local protocols [44].

Scene- management was another topic of interest to the participants. In this regard, Rosenzweig et al. 2012 stated that communication skills help the nurse to examine the patient and family's perception of illness or current events [45]. Fallowfield et al. 2003 stated that inadequate, confusing, or careless contact would lead to dissatisfaction among everyone involved. When bad news is given to individuals, the experience of receiving unpleasant news remains long after the initial encounter with the recipient [46]. Awareness of how the patient's family is treated and the type of behavior of the personnel with the patient's companions before the patient's death can be effective in accepting unpleasant news [47].

### BDN requirements

The concept of BDN requirements was another major subject in this study. The findings of this study show that experienced personnel and those who have faced several deaths are better at informing families about death. Public education and staff training, promote the culture of acceptance of unpleasant events by the people. It helps healthcare workers to announce BDN correctly. In Jones et al.'s 2008 study, the public has unrealistic expectations regarding CPR, citing this phenomenon as a result of incorrect expression of CPR results on TV and other sources of information. They recommended the use of TV as a source of information about cardiopulmonary resuscitation, personalized medical education, and the use of public programs on resuscitation for general education to improve CPR viewpoint [48]. Cook et al. (2002) also believed that announcing bad news to the bereaved family required special skills on the part of the staff. Unfortunately, there is little guidance or training on the approach to this very sensitive issue [49]. In our opinion, long-term planning and proper use of multimedia, radio, and television resources can be profitable in resolving this issue.

Another issue that participants viewed as a challenge was experienced in how to convey news of death to patients' companions. Wandkift et al. 2007 also stated that a fully trained staff in this field would be in a better position to handle the transfer of bad news [50]. Brown et al. also stated that there is evidence that inexperienced physicians are more affected by stress in disseminating bad news than experienced people [51]. The results of these studies are in line with the results of our study. Regarding the explanation of this case, it is notable that the medical staff is satisfied with their experience and not with other training that they have been familiarized within the college [52]. In addition to adding some university courses, designing simulation spaces to practice BDN can also be effective.

## Limitations

One of the limitations of our study was the COVID-19 issue, which causes restrictions on traffic and the non-cooperation of participants. Pre-hospital EMS staff may be reluctant to participate in the study due to impatience and intolerance of the interview process or the disclosure of their identity, or may not answer or answer some questions incorrectly. To ameliorate these problems, the researcher tried to make an acceptable connection and provide the necessary explanations to the research units, and they assured that the information would be completely confidential and that their answers would not be misused.

## Conclusion

This study aimed to explore the experience of pre-hospital EMS personnel in declaring the patient's death to families. During this process, five main themes emerged. BDN is affected by the stress of violence, the stress of family health, the stress of denial. Also, components such as individual characteristics, accident location, and type of death affect the way death is declared. The staff performed futile care and slow CPR to avoid tension until the dead person was transferred to the hospital. Staff should also consider scene safety, conditions of the family, scene management for BDN. For optimal BDN by EMS personnel, public education, training the staff, and having a death declaration experience are required.

## Supplementary Information

**Additional file 1.** 

## Data Availability

The datasets generated and/or analyzed during the current study are not publicly available due they are written in Persian and entered into MAXQUDA software, but are available from the corresponding author on reasonable request.

## Abbreviations

BDN: Braking Death News
CPR: Cardiopulmonary Resuscitation
KUMS: Kermanshah University of Medical Sciences
SD: Standard Deviation
EMS: Emergency Medical Service
