# Supplementary material for: The experience of pre-hospital emergency personnel in breaking death news: a phenomenological study
Source: BMC Nurs. 2022 May 25;21:127. doi: 10.1186/s12912-022-00899-x (PMC9130693; doi:10.1186/s12912-022-00899-x)
Supplement: Supplementary file 1 — Additional file 1. [file 12912_2022_899_MOESM1_ESM.docx]

**Consolidated criteria for reporting qualitative studies (COREQ): 32-item checklist**

Developed from:

Tong A, Sainsbury P, Craig J. Consolidated criteria for reporting qualitative research (COREQ): a 32-item checklist for interviews and focus groups. International Journal for Quality in Health Care. 2007. Volume 19, Number 6: pp. 349 – 357

| **No. Item** | **Guide questions/description** | **Reported on Page #** |
| --- | --- | --- |
| **Domain 1: Research team and reﬂexivity** |  |  |
| Personal Characteristics |  |  |
| 1. Inter viewer/facilitator | Which author/s conducted the inter view or focus group? | Page 4, lines 12-13 |
| 2. Credentials | What were the researcher’s credentials? E.g. PhD, MD | Page 1 |
| 3. Occupation | What was their occupation at the time of the study? | Page 1 |
| 4. Gender | Was the researcher male or female? | N/A |
| 5. Experience and training | What experience or training did the researcher have? | Page 4 lines 13-15 |
| Relationship with participants |  |  |
| 6. Relationship established | Was a relationship established prior to study commencement? | Page 4 line 4-5 |
| 7. Participant knowledge of the interviewer | What did the participants know about the researcher? e.g. personal goals, reasons for doing the research | Page 4, lines 4-5 |
| 8. Interviewer characteristics | What characteristics were reported about the inter viewer/facilitator? e.g. Bias, assumptions, reasons and interests in the research topic | Page 3 line 36  Page 4 lines 1-3 |
| **Domain 2: study design** |  |  |
| Theoretical framework |  |  |
| 9. Methodological orientation and Theory | What methodological orientation was stated to underpin the study? e.g. grounded theory, discourse analysis, ethnography, phenomenology, content analysis | Page 3 line 35  descriptive phenomenology |
| Participant selection |  |  |
| 10. Sampling | How were participants selected? e.g. purposive, convenience, consecutive, snowball | Page 4 line 2  purposeful sampling |
| 11. Method of approach | How were participants approached? e.g. face-to-face, telephone, mail, email | Page 4 line 6 |
| 12. Sample size | How many participants were in the study? | Page 5 line 9  thirteen |
| 13. Non-participation | How many people refused to participate or dropped out? Reasons? | N/A |
| Setting |  |  |
| 14. Setting of data collection | Where was the data collected? e.g. home, clinic, workplace | Page 4, lines 19-20 |
| 15. Presence of non-participants | Was anyone else present besides the participants and researchers? | N/A |
| 16. Description of sample | What are the important characteristics of the sample? e.g. demographic data, date | Page 5 , line 9-11 |
| Data collection |  |  |
| 17. Interview guide | Were questions, prompts, guides provided by the authors? Was it pilot tested? | Page 4, lines 6-12 |
| 18. Repeat interviews | Were repeat inter views carried out? If yes, how many? | N/A |
| 19. Audio/visual recording | Did the research use audio or visual recording to collect the data? | Page 4, line 14 |
| 20. Field notes | Were ﬁeld notes made during and/or after the inter view or focus group? | Page 4, line 11 |
| 21. Duration | What was the duration of the inter views or focus group? | Page 4 line 21 |
| 22. Data saturation | Was data saturation discussed? | Page 4, lines 12-13 |
| 23. Transcripts returned | Were transcripts returned to participants for comment and/or correction? | Page 5, lines 1,2 |
| **Domain 3: analysis and ﬁndings** |  |  |
| Data analysis |  |  |
| 24. Number of data coders | How many data coders coded the data? | Page 5, lines 13-14 |
| 25. Description of the coding tree | Did authors provide a description of the coding tree? | N/A |
| 26. Derivation of themes | Were themes identiﬁed in advance or derived from the data? | Page 5 lines 13- 22 |
| 27. Software | What software, if applicable, was used to manage the data? | MAQUDA-10  Page 4, line 24 |
| 28. Participant checking | Did participants provide feedback on the ﬁndings? | Page 5, line 2 |
| Reporting |  |  |
| 29. Quotations presented | Were participant quotations presented to illustrate the themes/ﬁndings? Was each quotation identiﬁed? e.g. participant number | Page 5-11 |
| 30. Data and ﬁndings consistent | Was there consistency between the data presented and the ﬁndings? | Discussion  Page 11-13 |
| 31. Clarity of major themes | Were major themes clearly presented in the ﬁndings? | All themes have been presented in details |
| 32. Clarity of minor themes | Is there a description of diverse cases or discussion of minor themes? | All subthemes have been presented in details |

**Once you have completed this checklist, please save a copy and upload it as part of your submission. When requested to do so as part of the upload process, please select the file type: Checklist. You will NOT be able to proceed with submission unless the checklist has been uploaded. Please DO NOT** **include this checklist as part of the main manuscript document. It must be uploaded as a separate file.**
